# Unsteady MHD Mixed Convection Slip Flow of Casson Fluid over Nonlinearly Stretching Sheet Embedded in a Porous Medium with Chemical Reaction, Thermal Radiation, Heat Generation/Absorption and Convective Boundary Conditions

**DOI:** 10.1371/journal.pone.0165348

**Published:** 2016-10-24

**Authors:** Imran Ullah, Krishnendu Bhattacharyya, Sharidan Shafie, Ilyas Khan

**Affiliations:** 1 Department of Mathematical Sciences, Faculty of Science, Universiti Teknologi Malaysia, 81310 UTM Johor Bahru, Johor, Malaysia; 2 Department of Mathematics, Institute of Science, Banaras Hindu University, Varanasi–221005, Uttar Pradesh, India; 3 College of Engineering, Majmaah University, Majmaah, 11952, Saudi Arabia; COMSATS Institute of Information Technology, PAKISTAN

## Abstract

Numerical results are presented for the effect of first order chemical reaction and thermal radiation on mixed convection flow of Casson fluid in the presence of magnetic field. The flow is generated due to unsteady nonlinearly stretching sheet placed inside a porous medium. Convective conditions on wall temperature and wall concentration are also employed in the investigation. The governing partial differential equations are converted to ordinary differential equations using suitable transformations and then solved numerically via Keller-box method. It is noticed that fluid velocity rises with increase in radiation parameter in the case of assisting flow and is opposite in the case of opposing fluid while radiation parameter has no effect on fluid velocity in the forced convection. It is also seen that fluid velocity and concentration enhances in the case of generative chemical reaction whereas both profiles reduces in the case of destructive chemical reaction. Further, increase in local unsteadiness parameter reduces fluid velocity, temperature and concentration. Over all the effects of physical parameters on fluid velocity, temperature and concentration distribution as well as on the wall shear stress, heat and mass transfer rates are discussed in detail.

## Introduction

Boundary layer flow over a stretching surface has several engineering and industrial applications, for example, in cooling bath, Aerodynamic extrusion, plastic sheet, metallurgical process, glass blowing, manufacturing of rubber and plastic sheets, crystal growing, the sheets are continuously stretched. During manufacturing, sheets are stretched continuously in order to achieve the desired thickness. It shows that final product depends upon the stretching and cooling rate of sheet. In the pioneer work of Sakiadis [1], he analyzed numerically the two dimensional flow over continuous stretched surface with the help of similarity transformations. Later on, many researchers have extended this work by considering different effects (one may refer to [2–9] and references therein). All these studies are restricted to linear stretching sheet, but the sheet velocity needs not be linear [3], it can be quadratic, exponential and non-linear. Keeping this in mind, Kumaran and Ramanaiah [10], Kechil and Ishak [11] analyzed viscous flow over quadratic stretching sheet. Magyari and Keller [12], Pramanik [13] investigated the heat and mass transfer characteristics in boundary layer flow over exponentially stretching sheet. Cortell [14], Hsiao [15], Jat et al. [16] analyzed combined heat transfer effects in two dimensional incompressible flow of viscous fluid cause due to nonlinearly stretching sheet.

All the above research dealt with steady stretching sheet, however in some case, the stretching sheet can be unsteady due to the impulsive motion of flat sheet. In this case, the flow field, heat and mass transfer are no longer steady and vary with time. The unsteadiness is because of the sudden change in wall velocity, free stream or wall temperature etc. Unsteady flow due to stretching sheet was examined by Pop and Na [17]. Later on, Sharidan et al. [18]explored the significance of variable wall temperature and variable heat flux in boundary layer flow over unsteady stretching surface using similarity transformations. Mukhopadhyay [19] studied the influence of thermal radiation on mixed convection flow of viscous fluid induced due to unsteady stretching sheet embedded in a porous medium. The effects of suction/injection on unsteady free convection flow of Newtonian fluid due to stretching sheet in the presence of chemical reaction has been developed by Chamkha et al. [20]. The unsteady stagnation point flow of viscous fluid caused by stretching sheet under the influence of slip condition was reported by Bhattacharyya et al. [21].

The impact of magnetohydrodynamic (MHD) and chemical reaction in heat and mass transfer flow has immense importance in many areas of engineering and industries. This phenomenon plays an important role in chemical industry, cooling of nuclear reactors, MHD power generation, MHD pumps, packed-bed catalytic reactor, formation and dispersion of fog, high speed plasma, cosmic jets, enhanced oil recovery, distribution of temperature and moisture over agriculture fields, cooling of nuclear reactors, manufacturing of ceramics, underground energy transport, food processing and cooling towers, etc. Takhar et al. [22] carried out an analysis for the chemical reaction effect on boundary layer flow over a stretching sheet under the influence of magnetic field. They found that magnetic field enhances the skin friction significantly. Motivated by this, Hsiao [23] analyzed hydromagnetic mixed convection flow along the wedge in the presence of suction and injection. The effect of thermal radiation and chemical reaction on unsteady free convection flow generated due to stretching sheet in the presence of magnetic field was investigated by Aurangzaib et al. [24]. Bhattacharyya et al. [25] discussed the unsteady boundary layer flow of electrically conducting fluid caused by stretching sheet under the influence of first order chemical reaction using similarity transformation. On the other hand the hydrodynamic slip effect on electrically conducting flow past a stretching sheet was successfully presented by Seini and Makinde [26]. The heat and mass transfer flow of hydromagnetic boundary layer flow towards stretching sheet with chemical reaction was reported by Mabood et al. [27].The influence of magnetic field on two dimensional flow of nanofluid with and without slip condition was discussed by Khan et al. [28] and Mohyud-Din et al. [29,30], respectively. Khan et al. [31] investigated two dimensional electrically conducting flow of nanofluid due to stretching sheet under the influence of convective boundary condition. The effect of first order chemical reaction two dimensional flow of viscous fluid in the presence and absence of magnetic field Khan et al. [32] and Mohyud-Din et al. [33]. Keeping in view of its application, the influence of thermal radiation on viscous flow of micropolar nanofluid in the presence of magnetic field was developed by Mohyud-din et al. [34].

On the other hand, non-Newtonian fluids has gained attraction due to its wide range application in various industries, such as design of solid matrix heat, nuclear waste disposal, chemical catalytic reactors, geothermal energy production, ground water hydrology, transpiration cooling, petroleum reservoirs, etc. These fluids are more complicated as compared to Newtonian fluids due to nonlinear relation between stress and strain rate. Several models have been proposed for the study of non-Newtonian fluids, but yet not a single model is developed that exhibits all properties of non-Newtonian fluids. In literature, the simplest model is the Maxwell model. Among numerous non-Newtonian fluids, there is another fluid known as Casson fluid. Casson fluid is a shear thinning fluid which is assumed to have an infinite viscosity at zero rate of shear, a yield stress below which no flow occurs and a zero viscosity at an infinite rate of shear. The Casson model also called rheological model was originally developed by Casson [35] for viscous suspension of cylindrical particles [36]. Common examples of Casson fluid are honey, jelly, soup, tomato sauce, concentrated fruit juices, etc. Also, it is the most appropriate rheological model for blood and chocolate. Furthermore, Casson fluid possesses yield stress and has great importance in polymer processing industries and biomechanics. The influence of thermal radiation on unsteady flow of Casson fluid caused by stretching sheet subjected to suction/blowing was presented by Mukhopadhyay [37]. Nadeem et al. [38] also analyzed the three dimensional hydromagnetic flow of Casson fluid in a porous medium. Numerical solutions of electrically conducting slip flow of Casson nanofluid generated during stretching sheet under the influence of convective boundary condition using similarity transformations were presented by Ibrahim and Makinde [39]. Motivated by its applications and interesting features, Benazir et al. [40] studied unsteady Casson flow past a vertical cone and flat sheet in the presence of magnetic field. Very recently, Oyelakin et al. [41] investigated unsteady electrically conducting flow of Casson nanofluid in the presence of slip and convective boundary conditions.

Motivated by the above study on steady and unsteady stretching sheet and its widespread engineering and industry application, the objective of present investigation is to study the effects of chemical reaction and thermal radiation on unsteady electrically conducting flow of Casson fluid towards nonlinearly stretching sheet saturated in a porous medium in the presence of slip and convective boundary conditions. The highly nonlinear partial differential equations are transformed into system of ordinary differential equations via suitable transformations and then numerically solved using Keller-box method [42]. The numerical and graphical results are obtained by developed an algorithm in MATLAB software. The developed algorithm for present problem is validated through comparison with previously published results and excellent accuracy is achieved. The physical behavior of pertinent parameters on fluid velocity, temperature and concentration are highlighted and discussed.

## Mathematical Formulation

**Fig 1:**
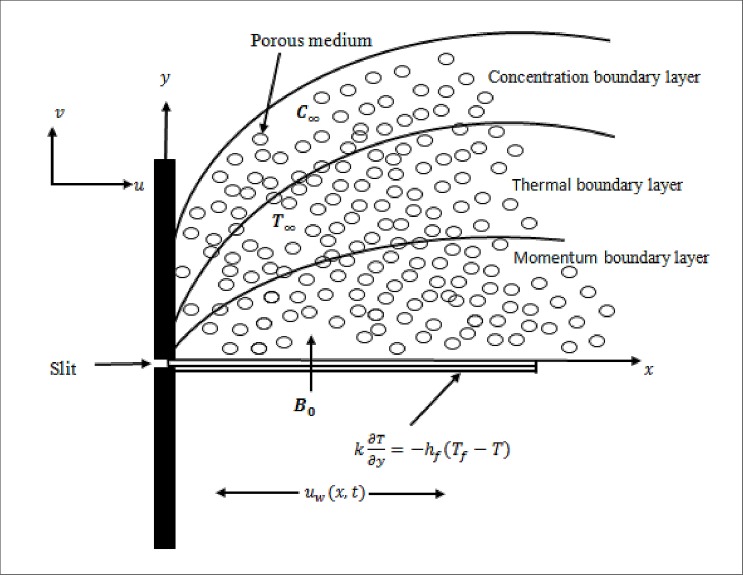
Physical sketch and coordinate system.

Consider hydromagnetic mixed convection flow of Casson fluid past an unsteady nonlinearly stretching sheet through porous medium under the influence of chemical reaction, thermal radiation, convective boundary conditions and partial slip. The *x*–axis is taken along the direction of stretching sheet and *y*–axis is perpendicular to the surface (see Fig 1). Initially at *t* ≤ 0 both the sheet and fluid are at rest and at the same temperature *T*_∞_ and concentration *C*_∞_. Suddenly, the sheet is stretched with the nonlinear velocity of the form *u*_*w*_(*x*,*t*) = *cx*^*n*^/(1 − *γt*), where *c*, *γ* ≥ 0 are constants, *t* is time and *n*(>0) represents the nonlinearly stretching sheet parameter such that, *n* = 1 corresponds to linear stretching sheet and *n* ≠ 1 represents nonlinear stretching sheet. Moreover, a magnetic field B(x,t)=B0x(n−1)/2(1−γt)−12 (being the function of *x* and *t*) is applied perpendicular to the stretching sheet with constant *B*_0_. Furthermore, it is also assumed that sheet wall is heated by temperature *T*_*f*_(*x*,*t*) and concentration *C*_*s*_(*x*,*t*). The temperature and nanoparticles concentration at free stream is *T*_∞_ and *C*_∞_, respectively.

The rheological equation of an isotropic and incompressible flow of Casson nanofluid can be written as (see Mukhopadhyay [37] and Oyelakin et al. [41])

$$\tau_{ij}=\{\begin{array}{l} \;2\left(\mu_{B}+p_{y}/\sqrt{2\pi }\right)e_{ij},\;\pi >\pi_{c},\\ 2\left(\mu_{B}+p_{y}/\sqrt{2\pi_{c}}\right)e_{ij},\;\pi <\pi_{c}. \end{array}$$ (1)

Here, *π* = *e*_*ij*_*e*_*ij*_ and *e*_*ij*_ is the (*i*,*j*)–*th* component of the deformation rate, *π* is the product of the component of deformation rate with itself, *π*_*c*_ is a critical value of this product based on the non-Newtonian model, *μ*_*B*_ is the plastic dynamic viscosity of the non-Newtonian fluid, and *p*_*y*_ is the yield stress of the fluid.

The governing equations for mixed convection flow of Casson fluid along with continuity equation are given as

$$\frac{\partial u}{\partial x}+\frac{\partial \upsilon }{\partial y}=0,$$ (2)

$$\frac{\partial u}{\partial t}+u\frac{\partial u}{\partial x}+\upsilon \frac{\partial u}{\partial y}=\nu \left(1+\frac{1}{\beta }\right)\frac{\partial^{2}u}{\partial y^{2}}-\left(\frac{\sigma B^{2}(x,t)}{\rho }+\frac{\nu \varphi }{k_{1}}\right)u\pm g\beta_{T}(T-T_{\infty })\pm g\beta_{C}(C-C_{\infty }),$$ (3)

$$\frac{\partial T}{\partial t}+u\frac{\partial T}{\partial x}+\upsilon \frac{\partial T}{\partial y}=\frac{k}{\rho c_{p}}\frac{\partial^{2}T}{\partial y^{2}}-\frac{1}{\rho c_{p}}\frac{\partial q_{r}}{\partial y}+\frac{\nu }{c_{p}}\left(1+\frac{1}{\beta }\right){\left(\frac{\partial u}{\partial y}\right)}^{2}+\frac{\sigma B^{2}\left(x,t\right)}{\rho c_{p}}u^{2}+\frac{Q\left(x,t\right)}{\rho c_{p}}\left(T-T_{\infty }\right),$$ (4)

$$\frac{\partial C}{\partial t}+u\frac{\partial C}{\partial x}+\upsilon \frac{\partial C}{\partial y}=D\frac{\partial^{2}C}{\partial y^{2}}-k_{c}\left(C-C_{\infty }\right).$$ (5)

In the above expressions *u* and *υ* denote the velocity components in *x* and *y* direction respectively, *ν* is kinematic viscosity, *σ* is the electrical conductivity, *β* is the Casson parameter, *ρ* is the fluid density, *φ* is the porosity, *k*_1_ = *k*_0_(1 − *γt*)/*x*^(*n*−1)^ is the variable permeability of porous medium, *g* is the gravitational force due to acceleration, *β*_*T*_ is the volumetric coefficient of thermal expansion, *β*_*C*_ the coefficient of concentration expansion, *k* is the thermal conductivity of the Casson fluid, *q*_*r*_ is the radiative heat flux, $Q\left(x,t\right)=\frac{Q_{0}x^{n-1}}{\left(1-\gamma t\right)}$ is heat generation/absorption coefficient, *D* is the mass diffusivity and *k*_*c*_ is the rate of chemical reaction.

The corresponding boundary conditions are written as follows:

$$t<0:\;u=\upsilon =0,\;T=T_{\infty },\;C=C_{\infty }\;\text{for any}\;x,y$$ (6)

$$t\geq 0:\;\begin{array}{l} u=u_{w}\left(x\right)+N_{1}\nu \left(1+\frac{1}{\beta }\right)\frac{\partial u}{\partial y},k\frac{\partial T}{\partial y}=-h_{f}\left(T_{f}-T\right)\\ D\frac{\partial C}{\partial y}=-h_{s}\left(C_{s}-C\right)\;\text{at}\;y=0 \end{array}\},$$ (7)

$$u\to 0,\;T\to T_{\infty },C\to C_{\infty }\;\text{as}\;y\to \infty .$$ (8)

Here N1(x,t)=N0x−(n−1)2(1−γt)12 denotes velocity slip factor with constant *N*_0_, hf(x,t)=h0x(n−1)2(1−γt)−12 and hs(x,t)=h1x(n−1)2(1−γt)−12 represents the convective heat and mass transfer with *h*_0_, *h*_1_ being constants, *T*_*f*_(*x*,*t*) = *T*_∞_ + *T*_0_*x*^*m*^(1 − *γt*)^−*m*^ in which *T*_0_ being reference temperature and *m* = 2*n* − 1 and *C*_*s*_(*x*,*t*) = *C*_∞_ + *C*_0_*x*^*m*^(1 − *γt*)^−*m*^ with *C*_0_ being reference concentration.

The expressions *u*_*w*_(*x*,*t*), *B*(*x*,*t*), *T*_*f*_(*x*,*t*), *C*_*s*_(*x*,*t*), *N*_*1*_(*x*,*t*), *h*_*f*_(*x*,*t*) and *h*_*s*_(*x*,*t*) are valid for *t* > *γ*^−1^.

The radiative heat flux *q*_*r*_ described according to Rosseland approximation is give as

$$q_{r}=\frac{-4\sigma^{*}}{3k_{1}{}^{*}}\frac{\partial T^{4}}{\partial y}$$ (9)

where σ* is the Stefan-Boltzmann constant and *k*_1_^*^ is the mean absorption coefficient. *T*^4^ can be expressed as linear function of temperature. By expanding *T*^4^ in a Taylor series about *T*_∞_ and neglecting higher terms, we can write

$$T^{4}≅4T_{\infty }^{3}T-3T_{\infty }^{4}.$$ (10)

Incorporating Eq (9) and Eq (10) in Eq (4), we obtain

$$\frac{\partial T}{\partial t}+u\frac{\partial T}{\partial x}+\upsilon \frac{\partial T}{\partial y}=\frac{k}{\rho c_{p}}\frac{\partial^{2}T}{\partial y^{2}}+\frac{16\sigma^{*}T_{\infty }^{3}}{3\rho c_{p}k_{1}{}^{*}}\frac{\partial^{2}T}{\partial y^{2}}+\frac{\nu }{c_{p}}\left(1+\frac{1}{\beta }\right){\left(\frac{\partial u}{\partial y}\right)}^{2}+\frac{\sigma B^{2}\left(x,t\right)}{\rho c_{p}}u^{2}+\frac{Q\left(x,t\right)}{\rho c_{p}}\left(T-T_{\infty }\right)$$ (11)

Now introduce the stream function *ψ* defined in its usual notation in terms of velocity, a variable *η* and the following transformations;

$$\psi =\sqrt{\frac{2\nu c}{\left(n+1\right)\left(1-\gamma t\right)}}x^{\frac{n+1}{2}}f(\eta ),\;\eta =\sqrt{\frac{(n+1)c}{2\nu \left(1-\gamma t\right)}}x^{\frac{n-1}{2}}y,\;\theta =\frac{T-T_{\infty }}{T_{f}-T_{\infty }},\;\phi =\frac{C-C_{\infty }}{C_{s}-C_{\infty }}$$ (12)

The system of Eqs (3–7) and Eq (11) take the following form

$$\left(1+\frac{1}{\beta }\right)f^{\prime \prime \prime }+ff^{\prime \prime }-\frac{2n}{n+1}{f^{\prime }}^{2}-\left(M+K\right)f^{\prime }+\lambda \left(\theta +N\phi \right)=A\left(\frac{2}{n+1}f^{\prime }+\frac{1}{n+1}\eta f^{\prime \prime }\right)$$ (13)

$$\left(1+\frac{4}{3}R_{d}\right)\theta^{\prime \prime }+\mathrm{Pr}f\theta^{\prime }-\frac{2\left(2n-1\right)}{n+1}\mathrm{Pr}f^{\prime }\theta +\mathrm{Pr}\left(1+\frac{1}{\beta }\right)Ec{\left(f^{\prime \prime }\right)}^{2}+\mathrm{Pr}MEc{f^{\prime }}^{2}+\mathrm{Pr}\varepsilon \theta =\mathrm{Pr}A\left(\frac{2\left(2n-1\right)}{n+1}\theta +\frac{1}{n+1}\eta \theta^{\prime }\right)$$ (14)

$$\frac{1}{Sc}\phi^{\prime \prime }+f\phi^{\prime }-\frac{2\left(2n-1\right)}{n+1}f^{\prime }\phi -R\phi =A\left(\frac{2\left(2n-1\right)}{n+1}\phi +\frac{1}{n+1}\eta \phi^{\prime }\right)$$ (15)

$$f^{\prime }(0)=1+\delta \left(1+\frac{1}{\beta }\right)f^{\prime \prime }\left(0\right),\;\theta^{\prime }(0)=-Bi_{1}\left[1-\theta \left(0\right)\right],\;\phi^{\prime }(0)=-Bi_{2}\left[1-\phi \left(0\right)\right]$$ (16)

$$f^{\prime }(\infty )=0,\;\theta (\infty )=0,\;\phi \left(\infty \right)=0$$ (17)

In the above expressions, *A*, *M*, *K*, λ=±GrxRex2, *N*, *δ*, Pr, *R*_*d*_, *Ec*, *ε*, *Bi*_1_, *Bi*_2_, *Sc* and *R* are the local unsteadiness parameter, magnetic parameter, porosity parameter,thermal buoyancy parameter (*λ* > 0 corresponds to assisting flow, *λ* = 0 indicates no convection and *λ* < 0 denotes the opposing flow)(*Gr*_*x*_ and Re_*x*_ being Grashof number and local Reynold number respectively), buoyancy ratio parameter, slip parameter, Prandtl number, radiation parameter, Eckert number, heat generation/absorption parameter, Biot numbers, Schmidt number and chemical reaction parameter, and are defined as

A=γxcxn,M=2σB02ρc(n+1),K=2νφk0(n+1)c,Grx=2gβT(Tf−T∞)x3ν2(n+1),Rex=xuwν,N=βC(Cs−C∞)βT(Tf−T∞),δ=N0(n+1)c2ν,Pr=μcpk,Rd=4σ∗T∞3αk1*,Ec=uw2cp(Tf−T∞),ε=Q0ρcp(n+1)c,Bi1=h0k[2νc(n+1)]12,Bi2=h1D[2νc(n+1)]12,Sc=νD,R=2νxkc(n+1)uw.

The wall skin friction, wall heat flux and wall mass flux, respectively, are defined by

$$\tau_{w}=\mu_{B}\left(1+\frac{1}{\beta }\right){\left[\frac{\partial u}{\partial y}\right]}_{y=0},\;q_{w}=-{\left(\left(\alpha +\frac{16\sigma^{*}T_{\infty }^{3}}{3k_{1}{}^{*}}\right)\frac{\partial T}{\partial y}\right)}_{y=0}\;\text{and}\;q_{s}=-D{\left(\frac{\partial C}{\partial y}\right)}_{y=0}.$$

The dimensionless skin friction coefficient $Cf_{x}=\frac{\tau_{w}}{\rho u_{w}^{2}}$, the local Nusselt number $Nu_{x}=\frac{xq_{w}}{\alpha (T_{f}-T_{\infty })}$ and local Sherwood number $Sh_{x}=\frac{xq_{s}}{D(C_{s}-C_{\infty })}$ on the surface along *x*-direction, local Nusselt number *Nu*_*x*_ and Sherwood number *Sh*_*x*_ are given by

$$\begin{array}{l} {\left(Re_{x}\right)}^{1/2}Cf_{x}\sqrt{\frac{2}{n+1}}=\left(1+\frac{1}{\beta }\right)f^{\prime \prime }(0),\;{\left(Re_{x}\right)}^{-1/2}Nu_{x}\sqrt{\frac{2}{n+1}}=-\left(1+\frac{4}{3}R_{d}\right)\theta^{\prime }(0),\\ {\left(Re\right)}^{-1/2}Sh_{x}\sqrt{\frac{2}{n+1}}=-\phi^{\prime }(0). \end{array}$$

## Numerical Scheme

The highly nonlinear governing Eqs (13–15) along with the associated boundary conditions (16) and (17) are solved numerically via Keller-box method. The details of this method can be found in the book of Cebeci and Bradshaw [42]. Like several other finite difference methods, the Keller-box method is very effective as it allow us to get the numerical solutions to system of nonlinear differential equations. Among many other numerical techniques, the finite difference method is more flexible for the reason that initial approximations control the convergence rate. The four main steps are involved to obtain the numerical solutions via Keller-box method, and are as follow:

1. Reduce Eqs (13–15) into a system of first order equations;
2. Write the difference equations using central differences;
3. Linearize the algebraic equations via Newton’s method, and write them in matrix-vector form; and
4. Finally, solve the obtained linear system by tridiagonal elimination technique.

It is worth mentioning that uniform step size of Δ*η* = 0.01 is found to be satisfactory in obtaining the numerical solutions for each profile with an error tolerance 10^−5^.

## Results and Discussion

In the present study, unsteady mixed convection flow of Casson fluid due to nonlinearly stretching sheet through porous medium under the influence of magnetic field and chemical reaction is explored. Moreover, the influence of partial slip and convective boundary conditions is also considered. Numerical computations are carried out for various values of local unsteadiness parameter *A*, Casson fluid parameter *β*, nonlinear stretching sheet parameter *n*, magnetic parameter *M*, porosity parameter *K*, thermal buoyancy parameter *γ*, concentration buoyancy ratio parameter *N*, Prandtl number Pr, radiation parameter *R*_*d*_, Schmidt number *Sc*, slip parameter *δ* and Biot numbers *Bi*_1_, *Bi*_2_.The present results are compared and validated in Tables (1–3) with some existing results in the available literature for limiting cases.

**Table 1:** Comparison of skin friction coefficient for different values of *β* and *M* when *n* = 1, *β* → ∞, *Bi*_1_ → ∞, *Bi*_2_ → ∞ and *A* = *M* = *K* = *λ* = *N* = *δ* = *R*_*d*_ = *Sc* = *R* = 0.

$\left(1+\frac{1}{\beta }\right)f^{\prime \prime }\left(0\right)$				
*β*	*M*	Nadeem et al. [37]	Oyelakin et al. [43]	Present results
∞	0	-1.0042	1.00000	1.0000
5		-1.0954	-1.09544	-1.0955
1		-1.4142	-1.41421	-1.4144
∞	10	-3.3165	3.31662	-3.3166
5		-3.6331	-3.63318	-3.6332
1		-4.6904	-4.69042	-4.6904
∞	100	-10.049	-10.04987	-10.0499
5		-11.0091	-11.00909	-11.0091
1		-14.2127	-14.21267	-14.2127

**Table 2:** Comparison of skin friction coefficient when *β* → ∞, *Bi*_1_ → ∞, *Bi*_2_ → ∞, Pr = 6.8 and *A* = *M* = *K* = *λ* = *N* = *δ* = *R*_*d*_ = *Sc* = *R* = 0.

$\left(1+\frac{1}{\beta }\right)f^{\prime \prime }\left(0\right)$				
*A*	Chamkha et al. [23]	Sharidan et al. [21]	Mukhopadhyay et al. [36]	Present results
0.8	-1.261512	-1.261042	-1.261479	-1.2610
1.2	-1.378052	-1.377722	-1.377850	-1.3777

**Table 3:** Comparison of −*θ*′(0) for different Pr with *n* = 1, *β* → ∞, *Bi*_1_ → ∞, *Bi*_21_ → ∞ and *A* = *M* = *K* = *λ* = *N* = *δ* = *R*_*d*_ = *Sc* = *R* = 0.

−*θ*′(0)				
Pr	Grubka and Bobba [4]	Aurangzaib et al. [30]	Mabood et al. [32]	Present results
0.72	0.8086	0.8086	0.8088	0.8088
1	1.0000	1.0000	1.0000	1.0000
3	1.9237	1.9237	1.9237	1.9237
10	3.7207	3.7207	3.7207	3.7208
100	12.2940	12.3004	-	12.3004

Tables 1 and 2 present the comparison of skin friction coefficient for different values of*β*, *M* and *A*, respectively, with the results of Nadeem et al. [37], Oyelakin et al. [43], Chamkha et al. [23], Sharidan et al. [21] and Mukhopadhyay et al. [36]. The results showed an excellent agreement. Table 3 describes the comparison of heat transfer rate for increasing values of Pr with the results of Grubka and Bobba [4], Aurangzaib et al. [30] and Mabood et al. [32], and revealed in a good agreement.

**Fig 2:**
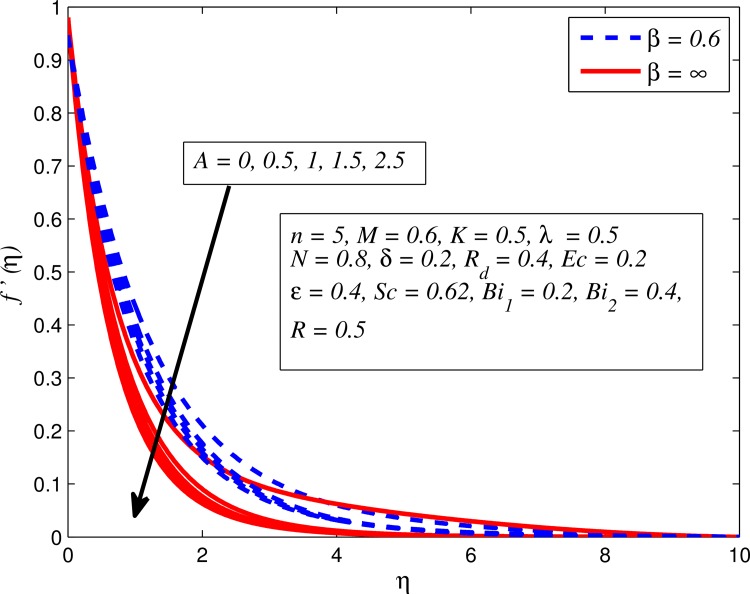
Effect of *A* on velocity for two different values of *β*.

**Fig 3:**
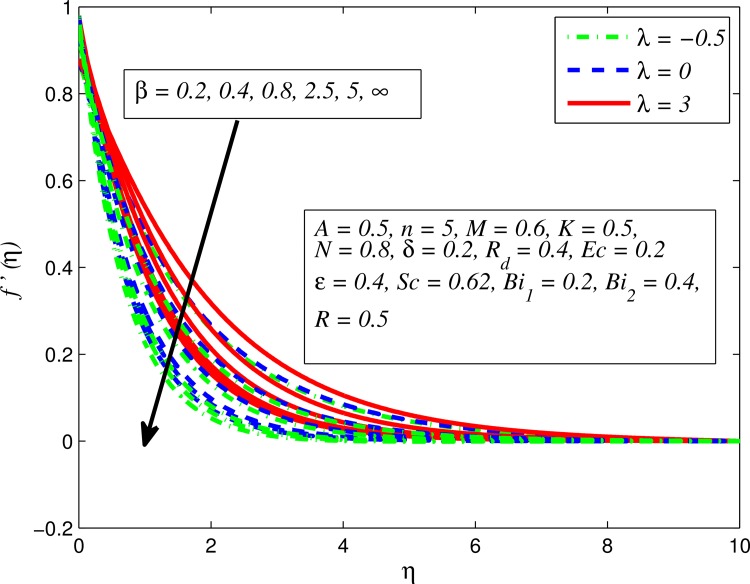
Effect of *β* on velocity for three different values of *λ*.

**Fig 4:**
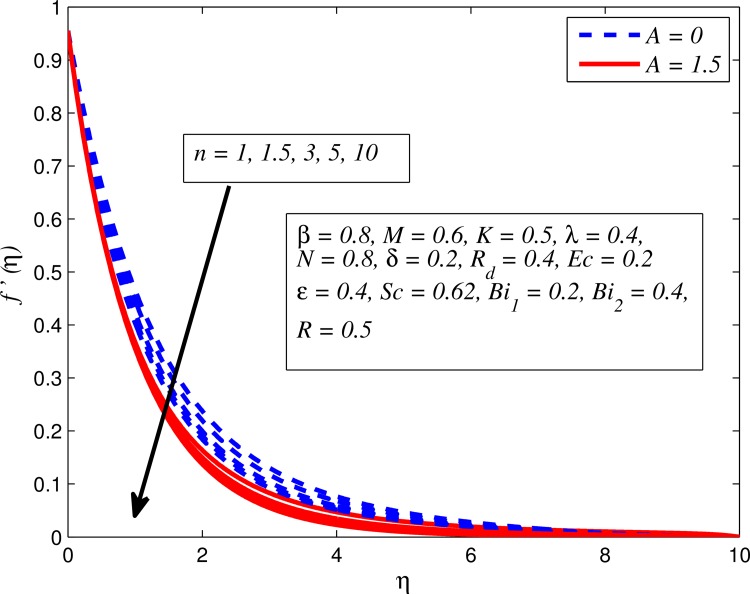
Effect of *n* on velocity for two different values of *A*.

**Fig 5:**
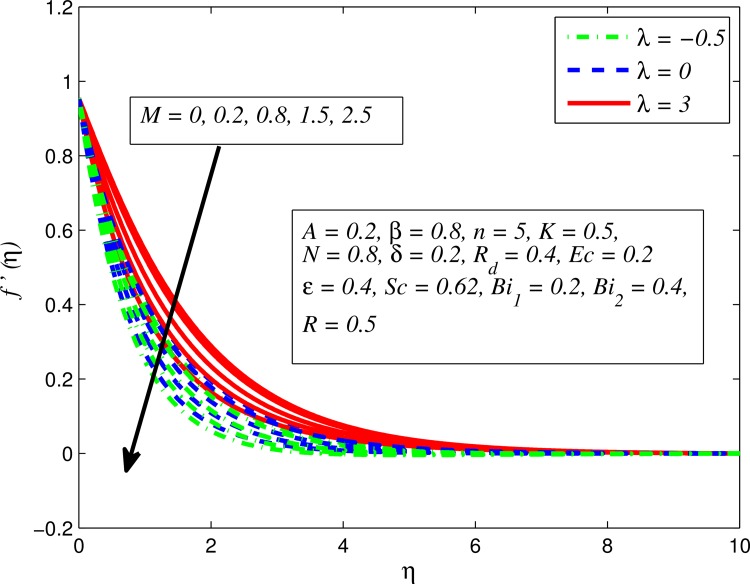
Effect of *M* on velocity for various values of *λ*.

**Fig 6:**
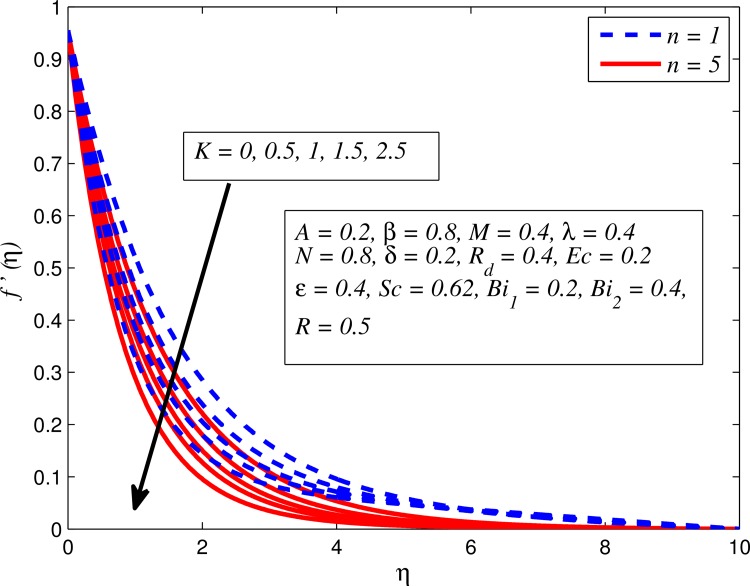
Effect of *K* on velocity for various values of *n*.

**Fig 7:**
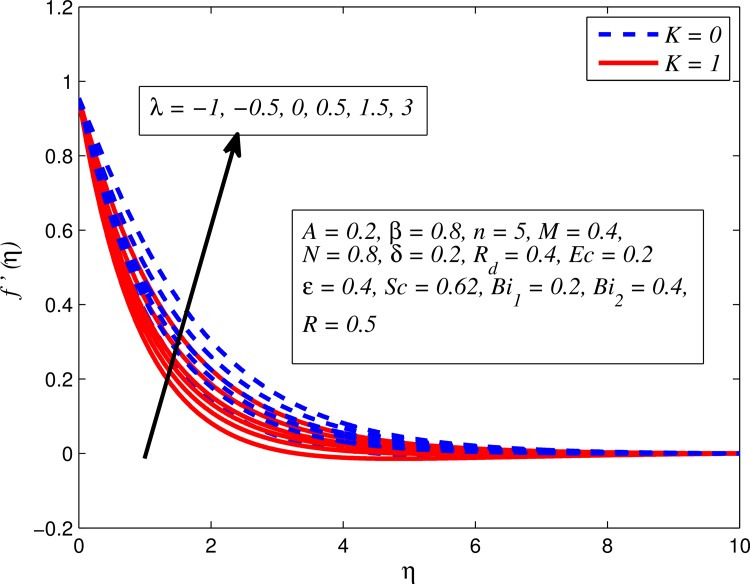
Effect of *λ* on velocity for two different values of *K*.

**Fig 8:**
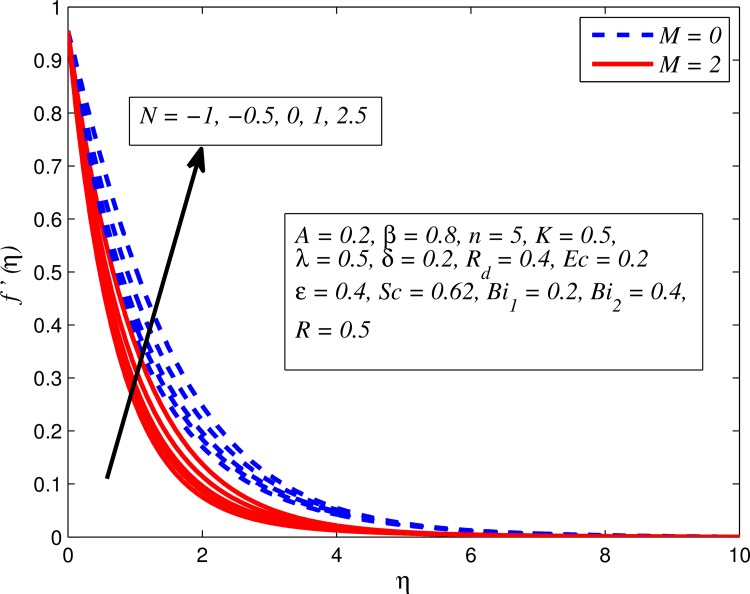
Effect of *N* on velocity for two different values of *M*.

**Fig 9:**
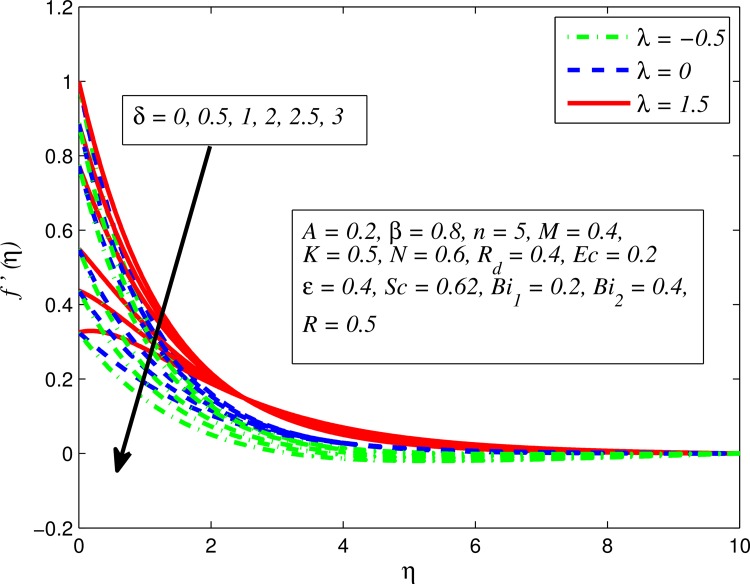
Effect of *δ* on velocity for different *λ*.

**Fig 10:**
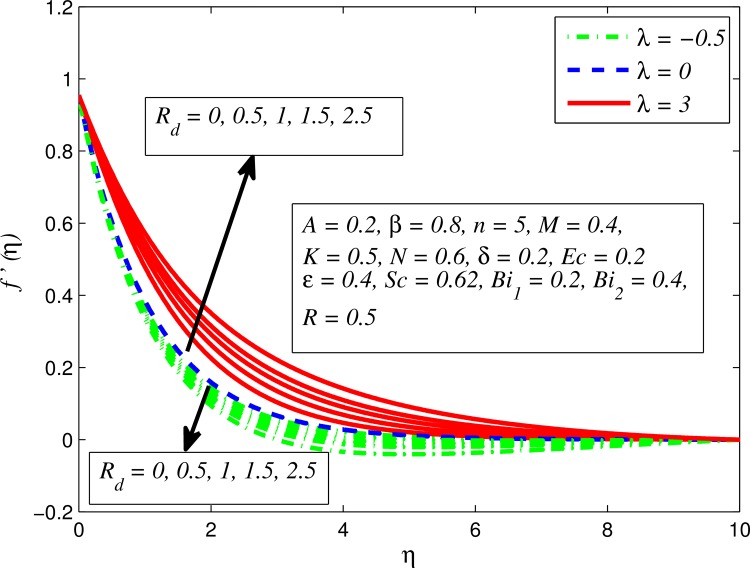
Effect of *R*_*d*_ on velocity for different *λ*.

**Fig 11:**
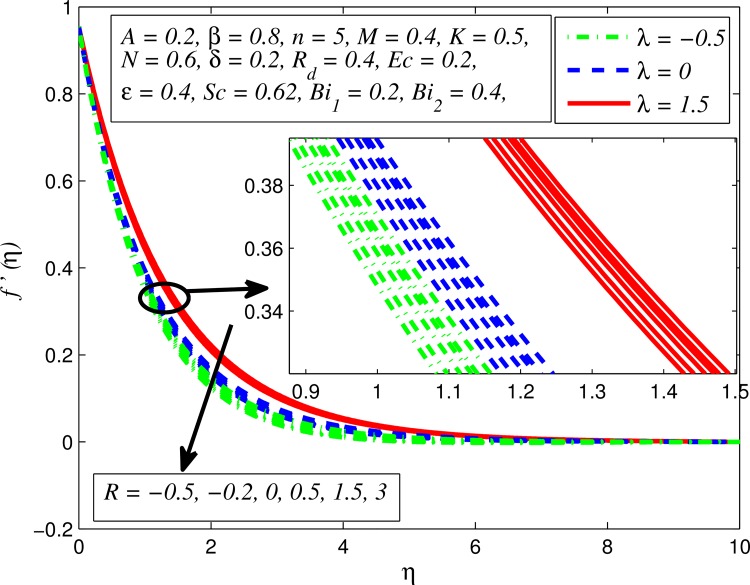
Effect of *R* on velocity for different *λ*.

Figs 2–11 show typical profile of velocity (*f*′(*η*)) for various values of *A*, *β*, *n*, *M*, *K*, *γ*, *N*, *δ*, *R*_*d*_ and *R*. Fig 2 depicts the effect of *A* on fluid velocity for *β* = 0.6 (Casson fluid) and *β* → ∞ (Newtonian fluid). It is found that fluid velocity reduces with increase in *A* for both fluids. It is also observed that larger amplitude of *A* thinning the boundary layer. Fig 3 presents that fluid velocity decelerates as *β* increases for all cases of *λ*. It is noticed that velocity boundary layer thickness reduces faster in the case of opposing flow (*λ* < 0) when *β* approaches towards infinity. Physically it makes sense because plasticity of fluid is higher as *β* goes higher and fluid experiences a resistance. Consequently, the velocity boundary layer becomes thinner for larger values of *β*. Fig 4 demonstrates the variation of *n* on velocity profile for both *A* = 0 and *A* ≠ 0. In both cases, fluid velocity falls with increase in *n*. However, the velocity boundary layer thickness rapidly reduces with *n* when *A* ≠ 0. Fig 5 clears that increasing values of *M* decrease the fluid velocity in all the three cases of *γ*. It is obvious, because the strength of magnetic field presents a resistance to the flow and results a decline in velocity boundary layer thickness. Moreover, a rapid decrease is seen in the fluid velocity with increase in *M* when *λ* < 0. Fig 6 reveals the effect of *K* on velocity profile for *n* = 1 and *n* > 1. It is seen that fluid velocity is lower with increase in *K*. Physically, the holes inside porous medium become wider as *K* increases, in this case fluid experiences a drag force which act opposite to the direction of flow and results a reduction in velocity boundary layer thickness. The influence of *λ* on velocity profile for *K* = 0 and *K* ≠ 0 is displayed in Fig 7. It is observed that fluid velocity is higher in the case of assisting flow (*λ* > 0) and lower in the case of opposing flow (*λ* < 0). This is physically realistic because in assisting flow buoyancy force dominates the viscous force which results an increment in the velocity, and in opposing flow the buoyancy force act opposite to the flow direction due to which the thickness of velocity boundary layer declines. On the basis of this phenomenon, it may be concluded that assisting flow thickens the velocity boundary layer whereas the velocity boundary layer becomes thinner in opposing flow. A similar kind of trend is observed for the effect of *N* on velocity profile in the absence and presence of magnetic field (see Fig 8). The influence of *δ* on velocity profile for all cases of *λ* is described in Fig 9. It is observed that for all the three case of *λ*, velocity is a decreasing function of *δ*. However, in the case of assisting flow the fluid velocity initially decrease in the vicinity of stretching sheet and then increases near the free stream as *δ* increased. This phenomenon can be explained as that increasing *δ* permitting more fluid to slip over the sheet due to which the flow near the sheet reduced and the slip effect towards the free stream is less pronounced. Fig 10 illustrates the variation of fluid velocity for various values of *R*_*d*_ when *λ* < 0, *λ* = 0 and *λ* > 0. It is interesting to notice that increasing values of *R*_*d*_ decline fluid velocity when *λ* < 0, has no effect when *λ* = 0 and enhances when *λ* > 0. Physically, it can be explained as that in assisting flow the convective heat transfer effect reduces with higher values of *R*_*d*_ due to which fluid get accelerated and opposite to this for opposing flow. Whereas, for *λ* = 0 the flow is force convective and then no effects of thermal radiation are found.

Next, Fig 11 shows the influence of the chemical reaction parameter *R* on velocity profile for various values *λ*. It is observed that fluid velocity slightly enhances in the case of generative chemical reaction (*R* < 0) and reverse effect in the case of destructive chemical reaction (*R* > 0). Hence velocity boundary layer slightly expands for generative chemical reaction and becomes thinner in case of destructive chemical reaction.

**Fig 12:**
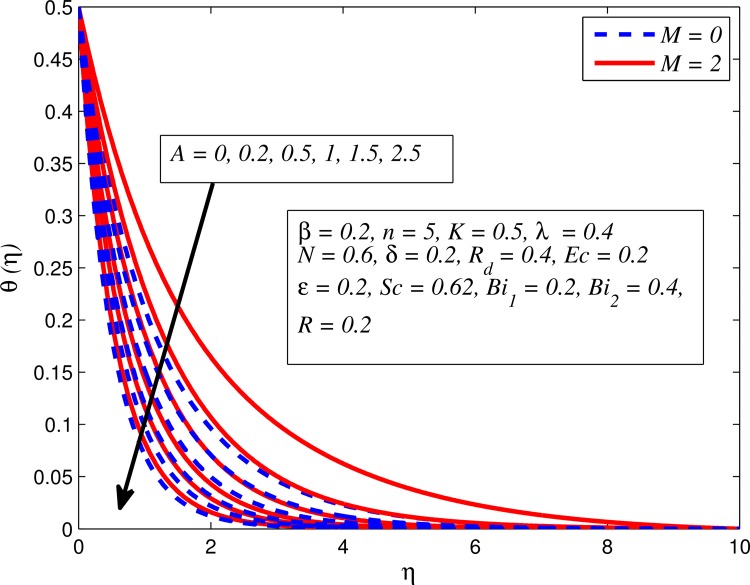
Effect of *A* on temperature for two different values of *M*.

**Fig 13:**
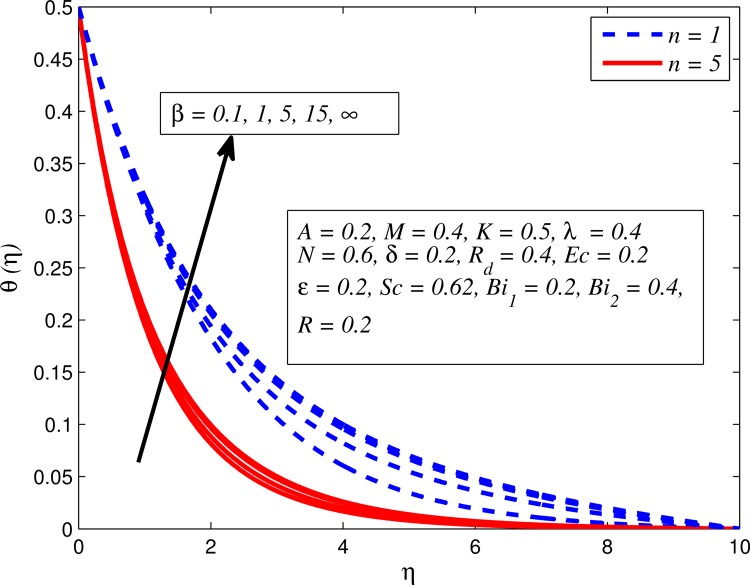
Effect of *β* on temperature for two different values of *n*.

**Fig 14:**
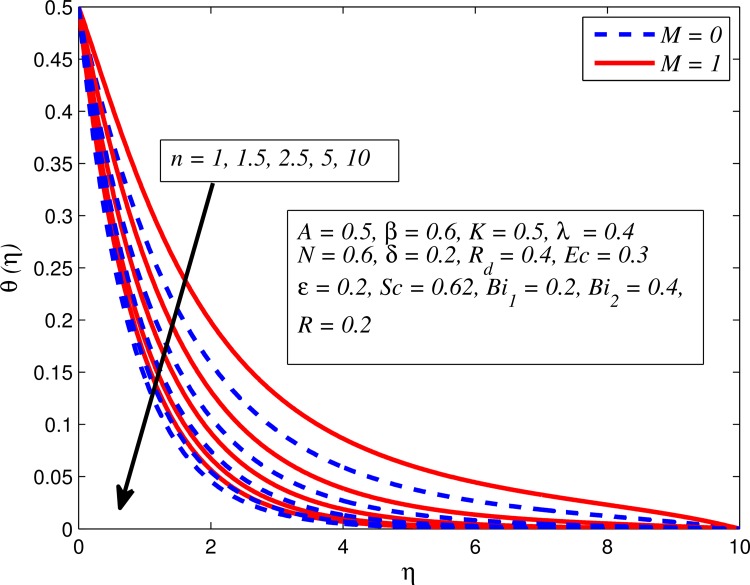
Effect of *n* on temperature for two different values of *M*.

**Fig 15:**
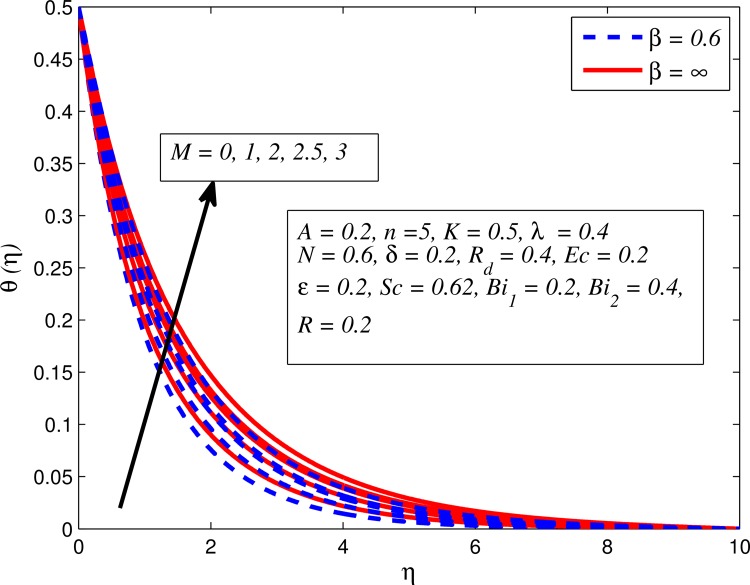
Effect of *M* on temperature for different *β*.

**Fig 16:**
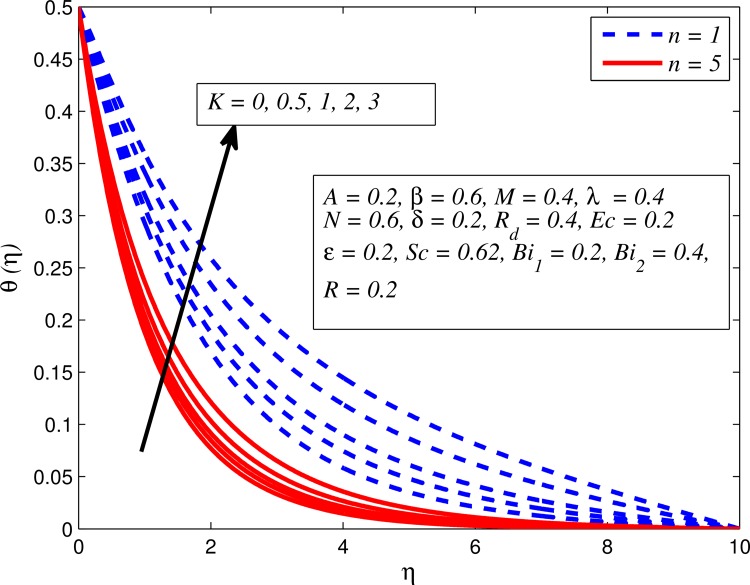
Effect of *K* on temperature for different *n*.

**Fig 17:**
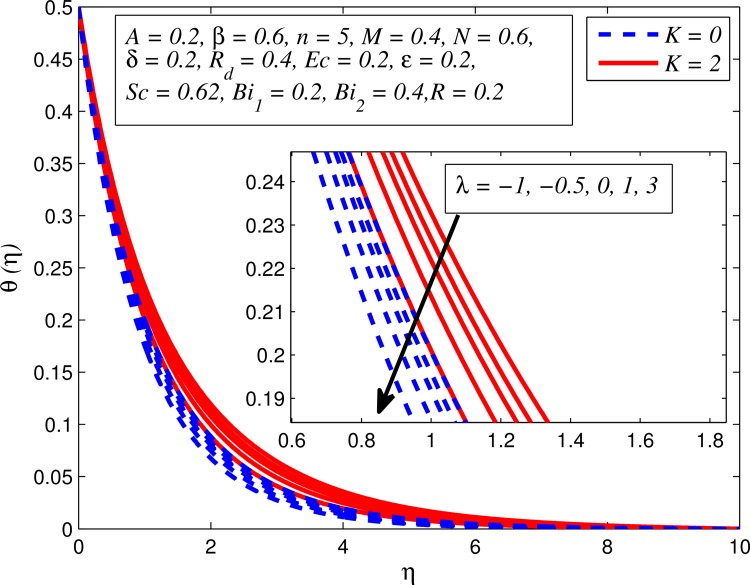
Effect of *λ* on temperature for different *K*.

**Fig 18:**
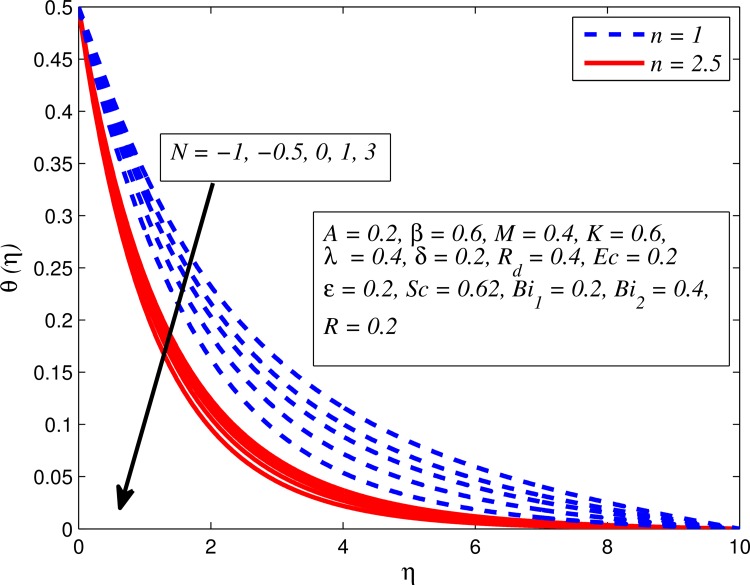
Effect of *N* on temperature for different *n*.

**Fig 19:**
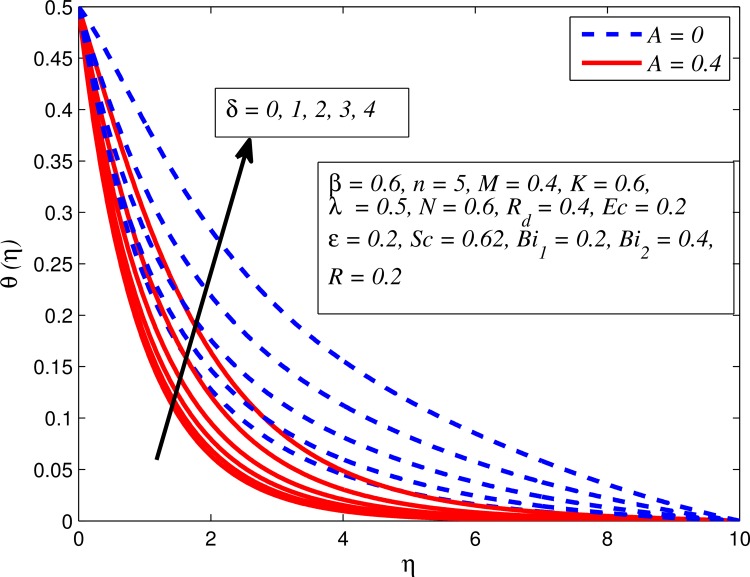
Effect of *δ* on temperature for different *A*.

**Fig 20:**
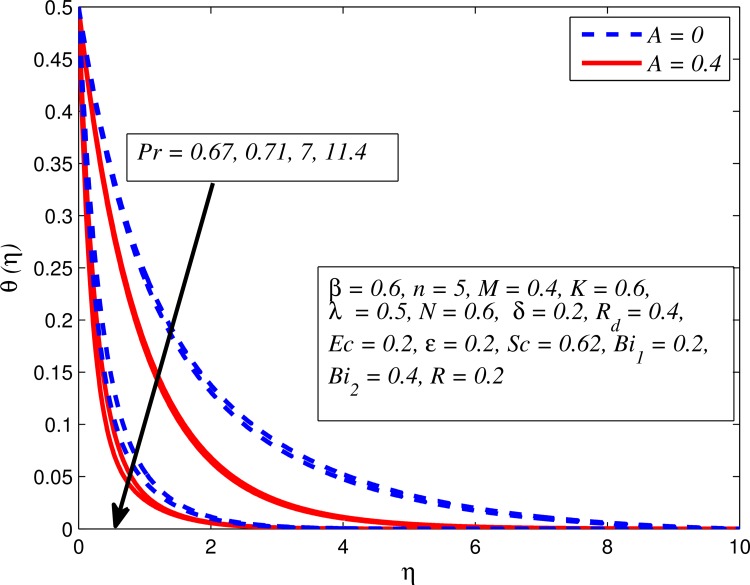
Effect of Pr on temperature for different *A*.

**Fig 21:**
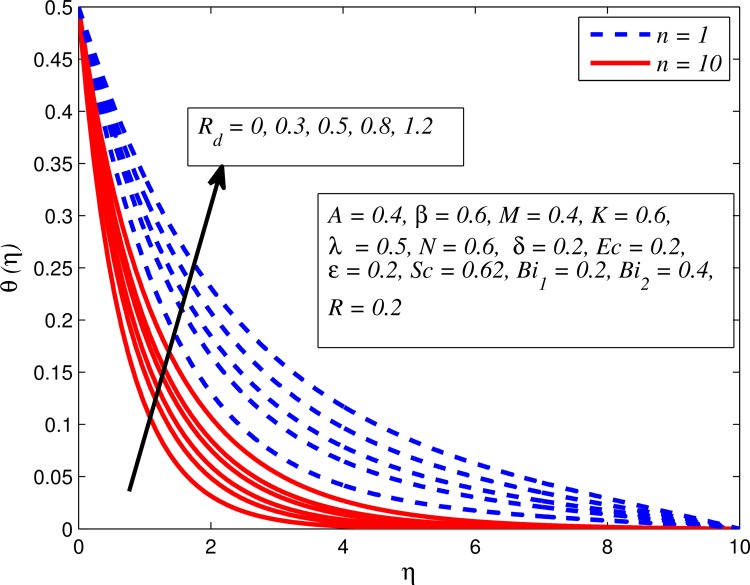
Effect of *R*_*d*_ on temperature for different *n*.

**Fig 22:**
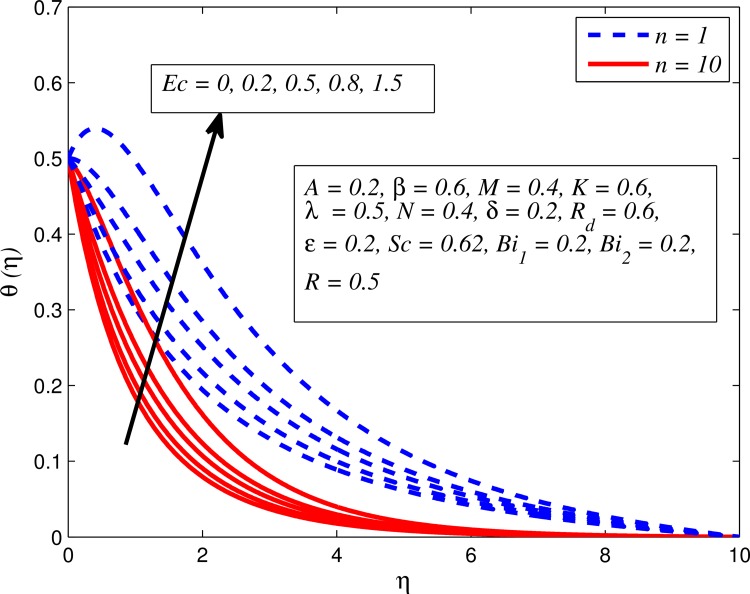
Effect of *Ec* on temperature for different *n*.

**Fig 23:**
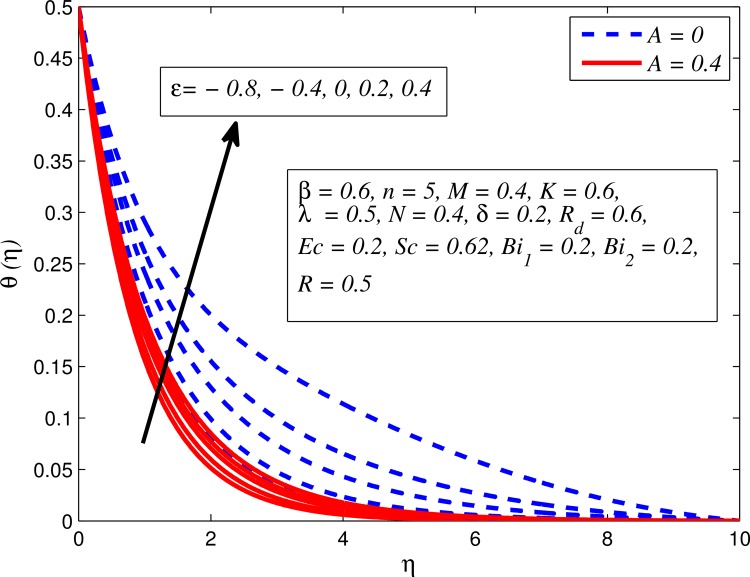
Effect of *ε* on temperature for different *A*.

**Fig 24:**
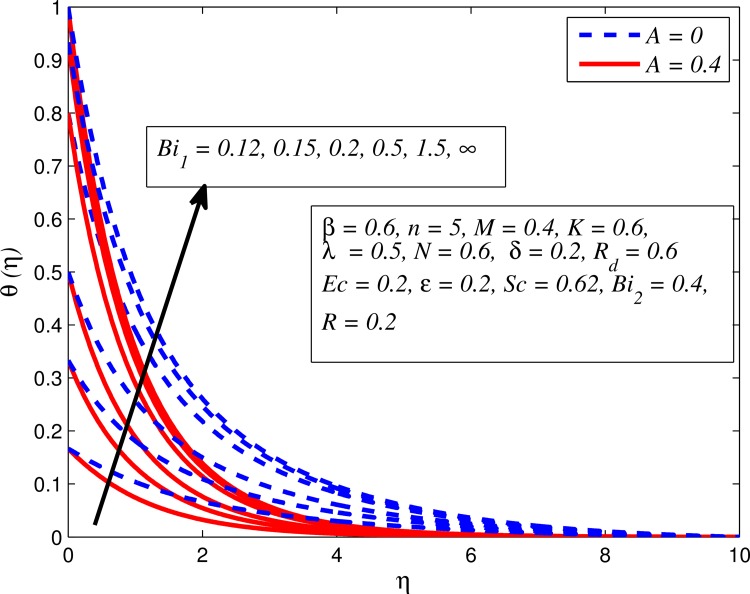
Effect of *Bi*_1_ on temperature for different *A*.

Figs 12–24 examined the variation of dimensionless temperature profile (*θ*(*η*)) for various values of *A*, *β*, *n*, *M*, *K*, *γ*, *N*, *δ*, Pr, *R*_*d*_, *Ec*, *ε* and *Bi*_1_. Fig 12 demonstrates the effect of *A* on temperature profile for *M* = 0 and *M* ≠ 0. It can be easily noticed that temperature decrease as *A* increased. Also, thermal boundary thickness in the case of *A* ≠ 0 is lower than *A* = 0. Clearly, Fig 13 reveals that increase in *β* lead to higher temperature across boundary region for both *n* = 1 and *n* ≠ 1. This phenomenon indicates that decrease in yield stress thickens thermal boundary layer. It is also observed that the influence of *β* is more pronounced for unsteady linear stretching sheet. The effect of *n* on temperature profile for *M* = 0 and *M* ≠ 0 is depicted in Fig 14. In both case the temperature is found to be reduced with increase in *n*. The thermal boundary layer becomes thinner when the sheet is stretched in a nonlinear way. Fig 15 elucidates the variation of temperature profile for various values of *M* when *β* = 0.6 (Casson fluid) and *β* → ∞ (Newtonian fluid). It is found that increasing values of *M* slightly enhance the temperature for both fluids. Since electromagnetic forces dominate viscous forces for higher values of *M*, it results in thickening thermal boundary layer. The influence of *K* on temperature profile for *n* = 1 and *n* ≠ 1 is plotted in Fig 16. The temperature is found to be increasing function of *K*. The influence of *K* is more prominent for steady flow and thermal boundary layer thicker as compared to unsteady flow. Fig 17 displays influenced of buoyancy parameter *λ* on the temperature. The thermal boundary layer becomes smaller due to increase in buoyancy parameter. It is caused since the buoyancy forces enhance temperature gradient and consequently the thermal boundary layer thickness reduces. A similar pattern is observed for the effect of *N* on temperature profile, i.e. thickness of thermal boundary layer reduces as *N* increases (see Fig 18). It is also noticed from this figure that temperature is more influenced by *N*in unsteady linear stretching sheet. The variation of temperature profile for various values of *δ* when *A* = 0 and *A* ≠ 0 is displayed in Fig 19. It is interesting to note that temperature is higher for larger values of *δ*. It is obvious because increasing *δ* allow more fluid to slip past a sheet, this results a rise in thermal boundary layer thickness. It is worth mentioning here that the effect is more pronounced in steady flow. Fig 20 portrays the effect of Pr (i.e. Pr = 0.67, 0.71, 7, 11.4 for Argon at 25°C, condensed air, water and water at 4°C) on temperature profile for both *A* = 0 and *A* ≠ 0. Prandtl number is the ratio of momentum diffusivity to thermal diffusivity. In both cases temperature reduces with increase in Pr. It is an agreement with the fact that higher values of Pr correspond to weaker thermal diffusivity which therefore thinning the thermal boundary layer. In addition, Pr is effectively used to control the both boundary thicknesses in heat transfer process. It is also noted from this figure that Argon at 25°C has thicker thermal boundary layer whereas water at 4°C has thinner boundary layer. The influence of *R*_*d*_ on temperature profile for *n* = 1 and *n* ≠ 1 is exhibited in Fig 21. It is observed that larger values of *R*_*d*_ rise the temperature significantly. Physically, it is true due to the fact that higher values of *R*_*d*_ lead to large radiation effect and thereby enhancing the thickness of thermal boundary layer. In the work [43] similar temperature pattern were obtained. Fig 22 portrays the variation of dimensionless temperature profile for various values of *Ec* when *n* = 1 and *n* ≠ 1. It can be easily noticed from this figure that temperature is higher for larger values of *Ec*. It is well known that viscous dissipation generates heat due to frictional heating between the fluid particles and this extra heat lead to temperature and associated boundary layer thickness enhancement. The effect of *ε* on dimensional temperature for *A* = 0 and *A* ≠ 0 is shown in Fig 23. It is worth mentioning here that *ε* > 0 represent heat generation and *ε* < 0 corresponds to heat absorption. Clearly, the existence of heat generation in the boundary layer leads to enhance the energy level. Consequently, thickens thermal boundary in the vicinity of sheet. On the other hand, opposite trend is observed in the presence of heat absorption, i.e. thermal boundary layer becomes thinner. It is also noticeable from this figure that the effect is more pronounced for steady flows. Fig 24 examined the effect of *Bi*_1_ on temperature profile for both *A* = 0 and *A* ≠ 0. It is noteworthy that *Bi*_1_ << 1 shows that temperature field is uniform inside the surface whereas *Bi*_1_ >> 1 indicates the non-uniformity of temperature field inside the body surface. Biot number *Bi*_1_ is the ratio of internal thermal resistance of the sheet to the boundary layer thermal resistance of the hot fluid at the bottom of the surface. In both cases, temperature is an increasing function of *Bi*_1_. The reason behind this is that strength of *Bi*_1_ increases the convection at the surface and thereby enhances the surface temperature.

**Fig 25:**
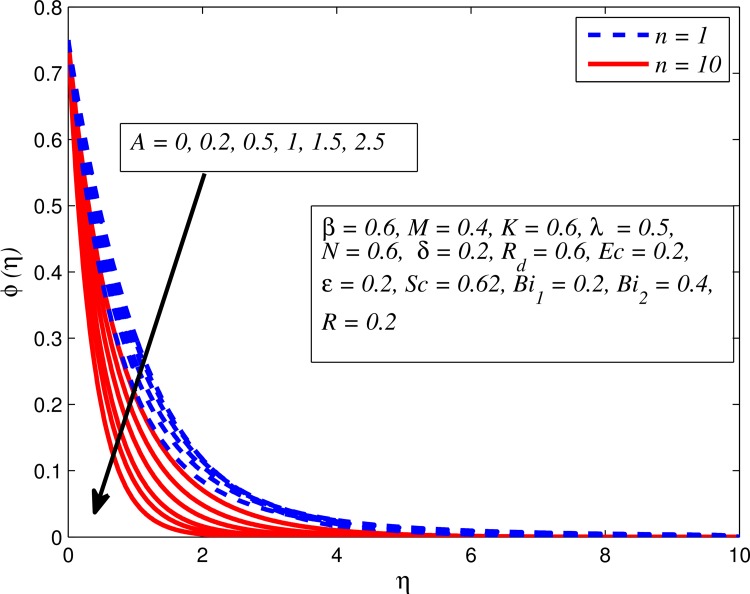
Effect of *A* on concentration for different *n*.

**Fig 26:**
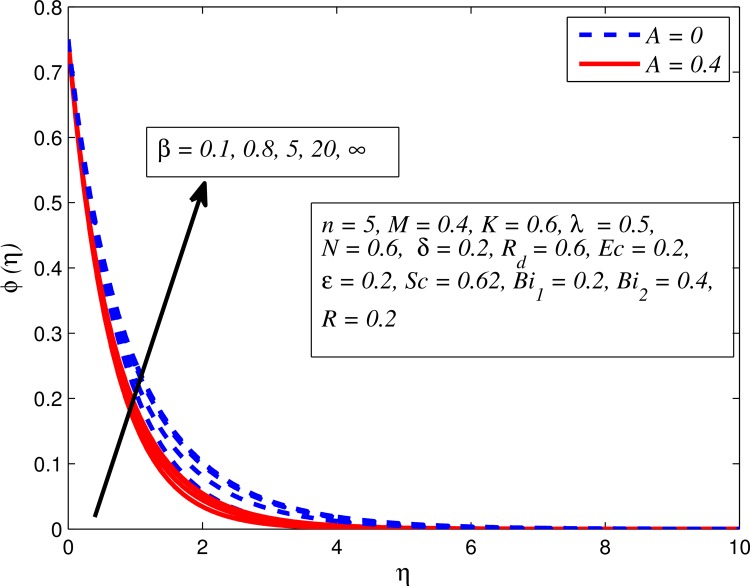
Effect of *β* on concentration for different *A*.

**Fig 27:**
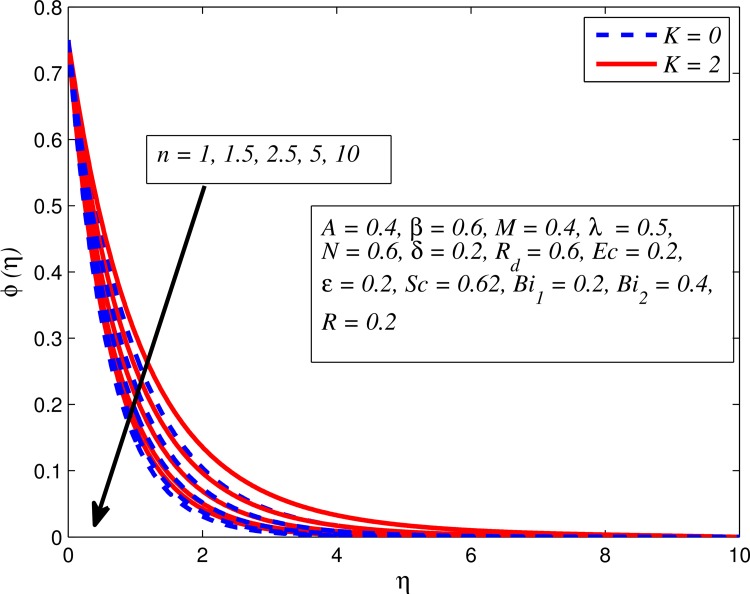
Effect of *n* on concentration for different *K*.

**Fig 28:**
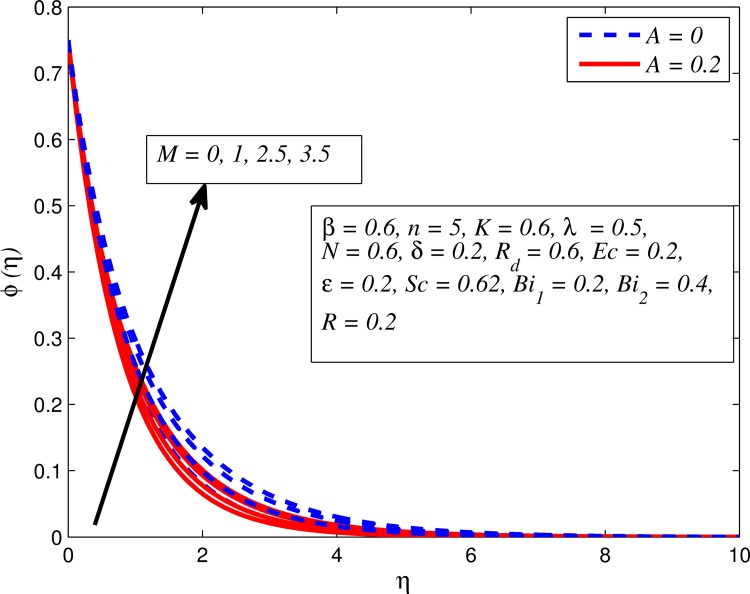
Effect of *M* on concentration for different *A*.

**Fig 29:**
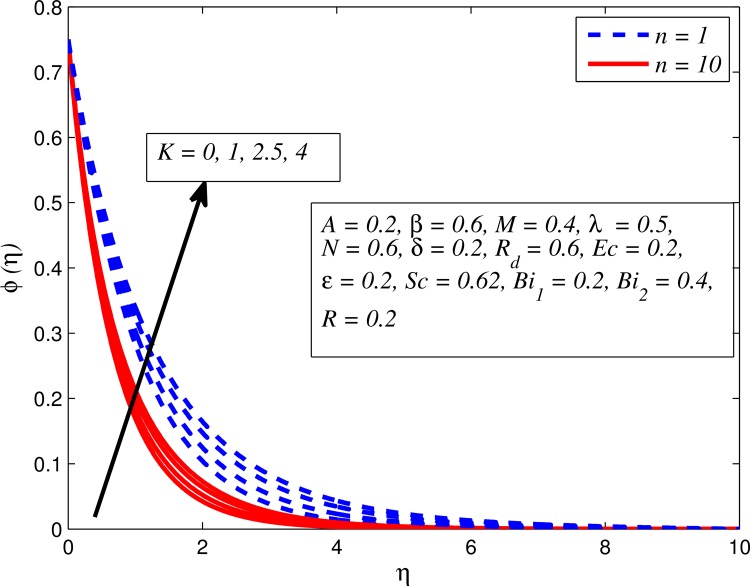
Effect of *K* on concentration for different *n*.

**Fig 30:**
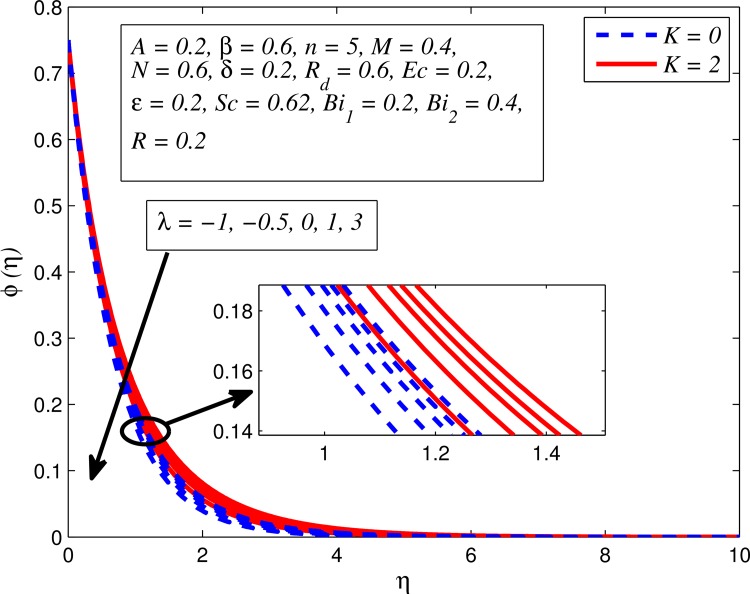
Effect of *λ* on concentration for different *K*.

**Fig 31:**
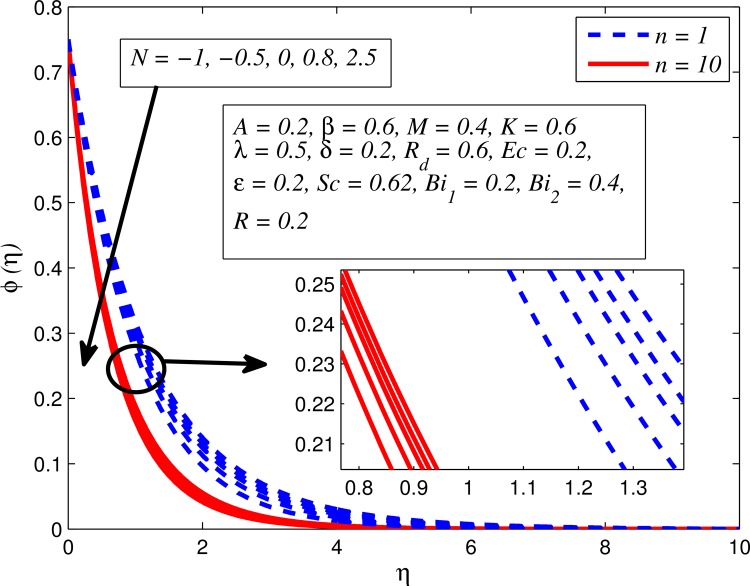
Effect of *N* on concentration for different *n*.

**Fig 32:**
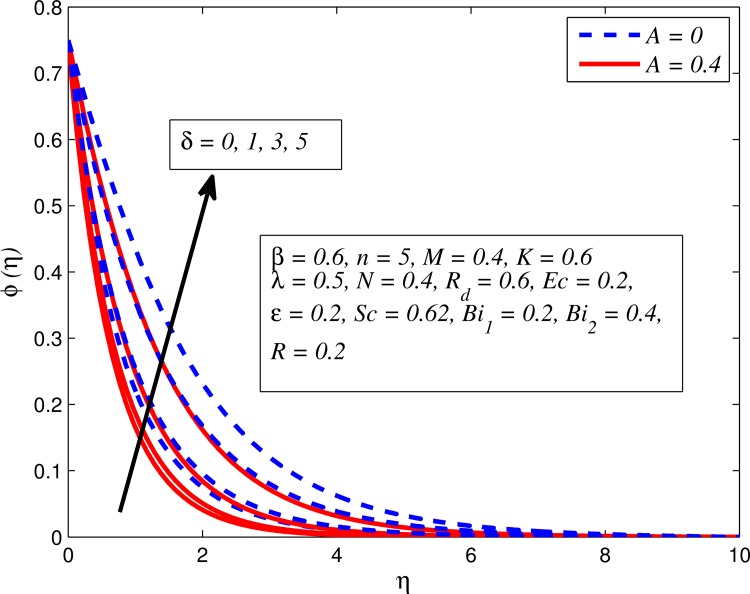
Effect of *δ* on concentration for different *A*.

**Fig 33:**
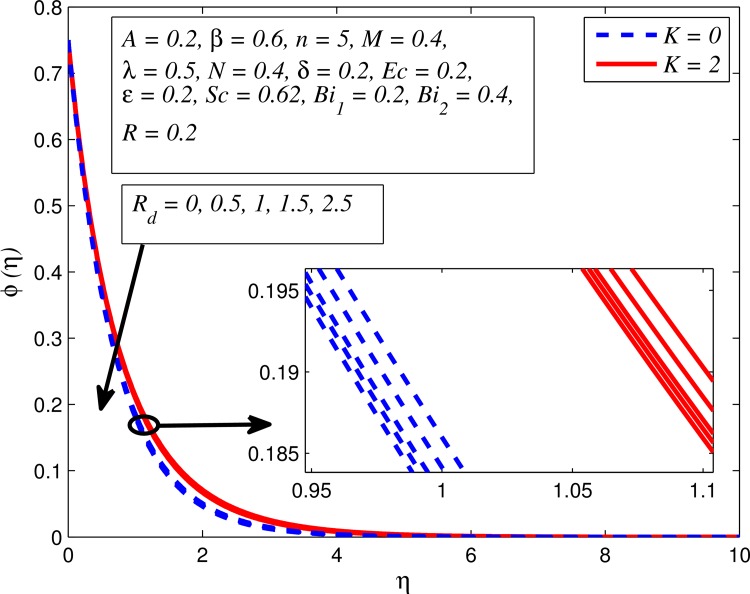
Effect of *R*_*d*_ on concentration for different *K*.

**Fig 34:**
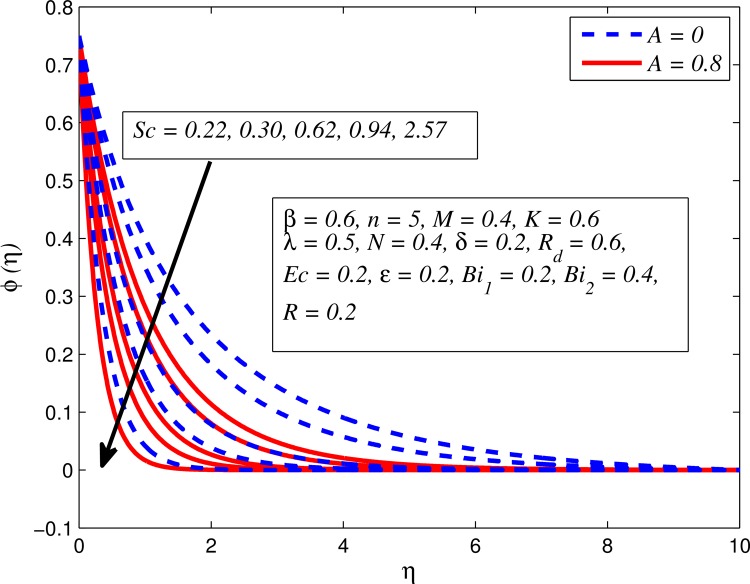
Effect of *Sc* on concentration for different *A*.

**Fig 35:**
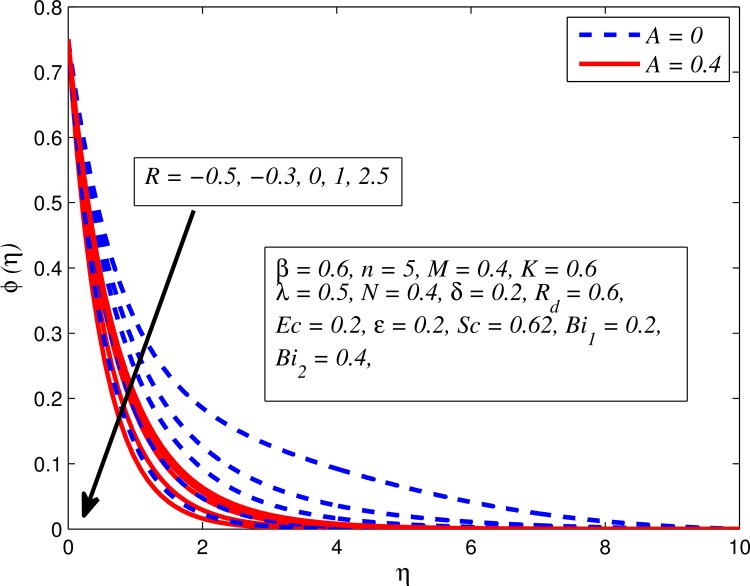
Effect of *R* on concentration for different *A*.

**Fig 36:**
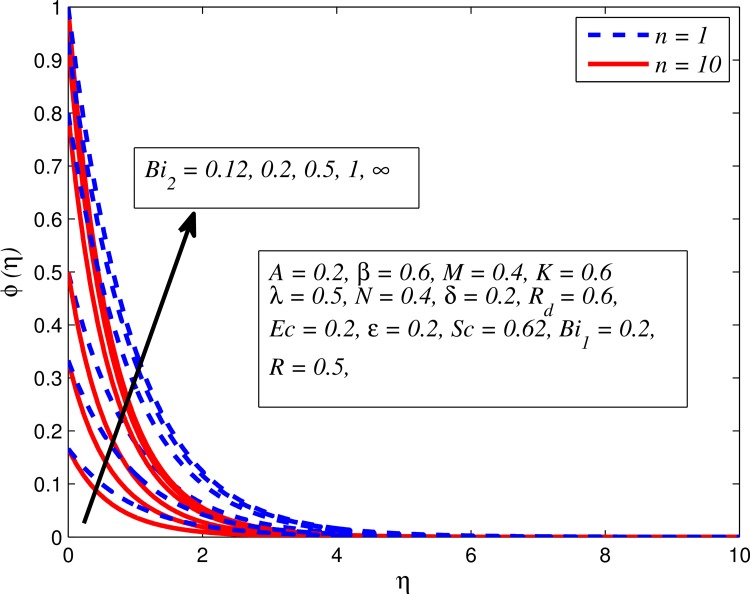
Effect of *Bi*_2_ on concentration for two different *n*.

Figs 25–36 illustrate the variation of concentration profile (*ϕ*(*η*)) for various values of *A*, *β*, *n*, *M*, *K*, *γ*, *N*, *δ*, Pr, *R*_*d*_, *Sc*, *R* and *Bi*_2_, respectively. Fig 25 reveals the variation of concentration profile for different value of *A* when *n* = 1 and *n* ≠ 1. This figure clearly shows that increasing values of *A* effectively reduces the concentration boundary layer thickness. It is also noticed that effect of *A* on concentration is more significant when *n* ≠ 1 (for nonlinearly stretching sheet). Fig 26 demonstrates that concentration distribution is higher for larger values of *β* in both cases of *A* = 0 and *A* ≠ 0. It is also noticed that the effect is more pronounced for steady flows. Fig 27 illustrates that concentration falls as *n* increases in both cases of *K* = 0 and *K* ≠ 0. The reduction in concentration boundary layer thickness is found for non-linearly stretching sheet. The influence of *M* on concentration profile for *A* = 0 and *A* ≠ 0 is depicted in Fig 28. It is noticeable that concentration distribution related boundary layer thickness enhance with increase in *M*. The reason already explained in Fig 5, i.e. strength of magnetic field enhances the Lorentz force which therefore reduces the velocity and results an increase in concentration boundary layer thickness. It is also observed that the effect of *M* is large when *A* = 0 (steady flow). A similar behavior of concentration profile is noted for increasing values of *K* when *n* = 1 and *n* ≠ 1 (see Fig 29). It is also found from this figure that the effect is more pronounced in linear stretching sheet. From Fig 30, it is noticed that concentration distribution is a decreasing function of *λ* for both *K* = 0 and *K* ≠ 0. However, the effect is very small due to the reason that the buoyancy term is only coupled with momentum equation. A same kind of effect has been observed for increasing values of *N* on concentration profile (see Fig 31). The influence of *δ* on concentration distribution for *A* = 0 and *A* ≠ 0 is displayed in Fig 32. This figure reveals that in both cases concentration and associated boundary layer thickness rise as *δ* increases. Since a bulk of fluid is slipping over the sheet which reduces the flow field and thereby thickening the concentration boundary layer. It is also noticed from this figure that concentration is more influenced by *δ* in the case of steady flow. Fig 33 clears that concentration slightly reduces with increase in *R*_*d*_. The reason behind this phenomenon is that stronger *R*_*d*_ lead to enhance the fluid velocity as well as temperature, and therefore concentration boundary layer becomes thinner. Fig 34 discusses the influence of *Sc* (i.e. *Sc* = 0.22,0.30,0.62,0.94,2.57 for hydrogen, helium, water vapor, hydrogen sulphide and Propyl Benzene) on concentration distribution when *A* = 0 and *A* ≠ 0. In both cases, the concentration distribution reduces with increase in *Sc*. Physically, larger values of *Sc* correspond to low mass diffusivity which leads to a thinning of the concentration boundary layer. The influence of *Sc* on fluid velocity and temperature is very small because the physical parameter arises only in concentration equation and is not displayed here for the sake of brevity. Clearly, Fig 35 describes that concentration rises in generative chemical reaction (*R* < 0) and opposite to this is observed in the case of destructive chemical reaction (*R* > 0). The physical reason behind this behavior is that strength of chemical reaction alters the diffusion rates. It is also noticeable that the effect is major for steady flow. The variation of concentration profile for different values of *Bi*_2_ when *n* = 1 and *n* ≠ 1 is portrayed in Fig 36. It can be easily seen that increasing values of *Bi*_2_ enhances the concentration distribution for both *n* = 1 and *n* ≠ 1. Since the concentration distribution is driven by temperature field. Hence, higher values of *Bi*_2_ would promote a deeper penetration of the concentration.

**Fig 37:**
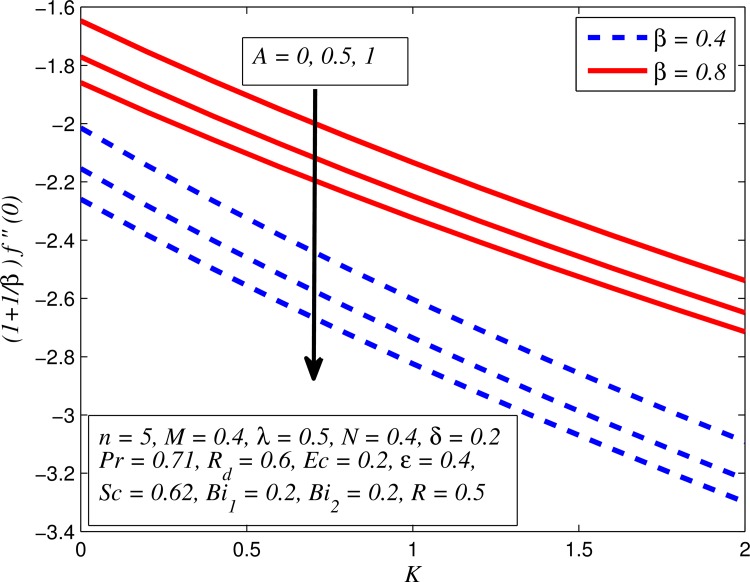
Variation of skin friction coefficient for various values of *A*, *β*, and *K*.

**Fig 38:**
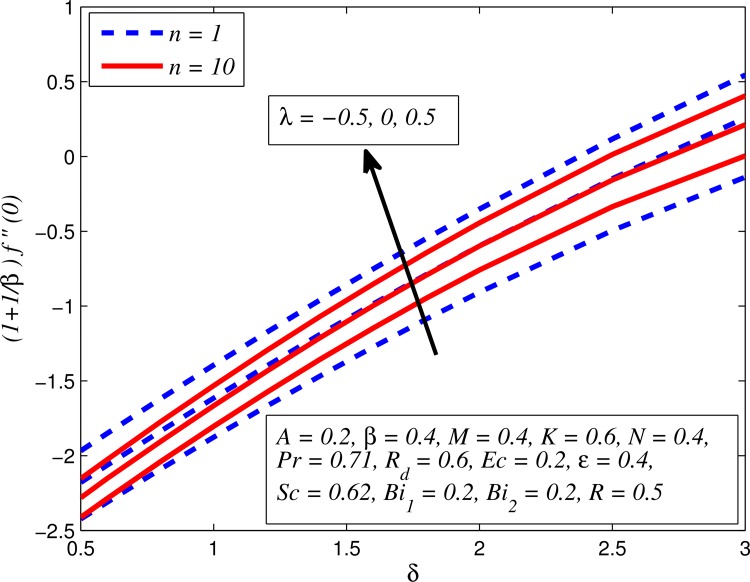
Variation of skin friction coefficient for various values of *λ*, *n* and *δ*.

**Fig 39:**
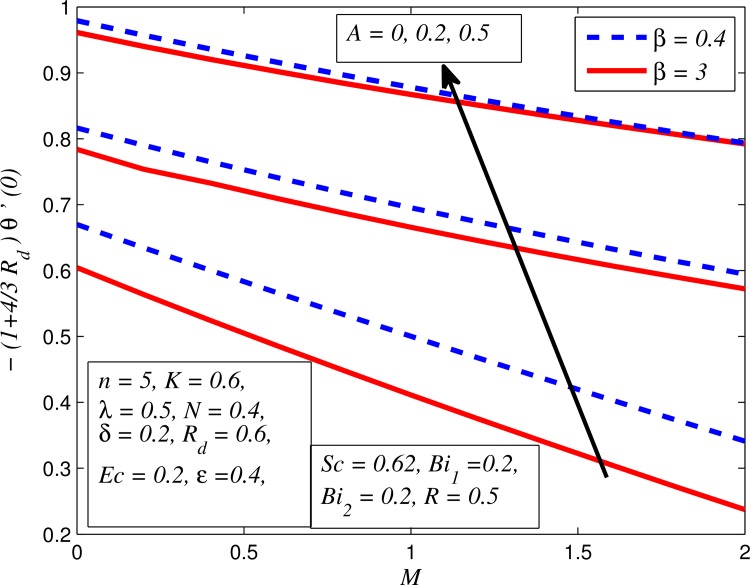
Variation of Nusselt number for various values of *A*, *β* and *M*.

**Fig 40:**
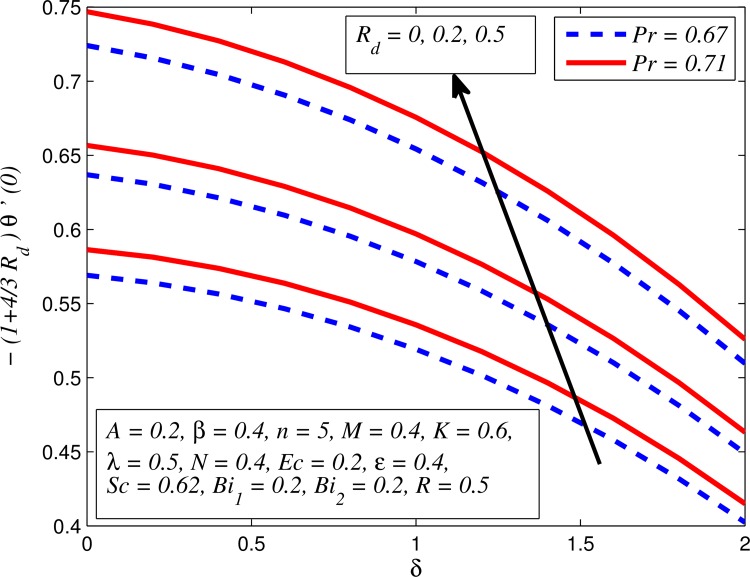
Variation of Nusselt number for various values of *R*_*d*_, *δ* and Pr.

**Fig 41:**
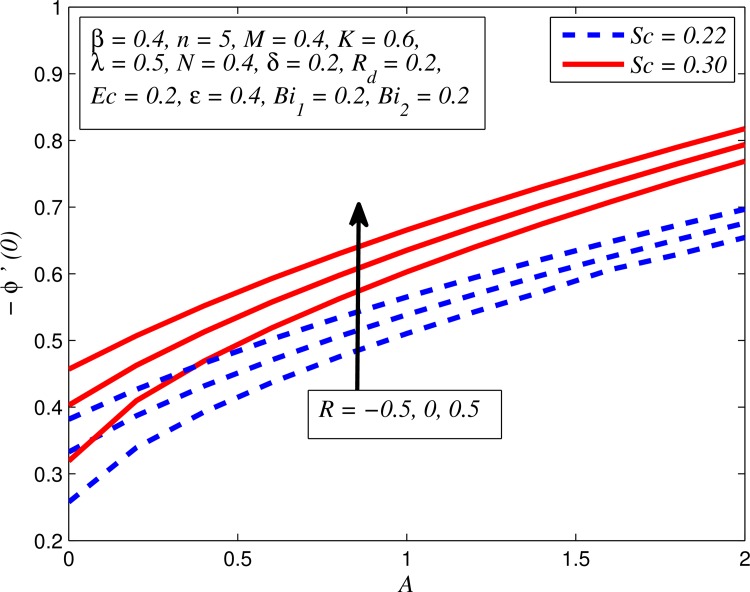
Variation of Sherwood number for various values of *Sc*, *A* and *R*.

Figs 37–41 examined the variation of skin friction coefficient, local Nusselt number and local Sherwood number for different values of *A*, *β*, *K*, *δ*, *n*, Pr, *R*_*d*_, *Sc*, *γ* and *R*. Fig 37 illustrates the variation of skin friction coefficient for various values of *A*, *β* and *K*. It is found that increasing values of *A* and *K* causes reduction in the wall shear stress whereas increasing values of *β* enhances the friction factor. Since the strength of permeability and unsteadiness in the flow decrease the flow velocity due to which the velocity boundary layer thickness reduces and consequently the enhancement of wall shear stress occurs. It is also noticed that wall shear stress is negative for all values of *A*, *β* and *K* which reveals that the fluid experiences a drag force from the stretching surface and positive implies the lift force. The influence of skin friction coefficient for different values of *λ*, *n* and *δ* is exhibited in Fig 38. This figure shows that wall shear stress enhances with increase in *λ* and *δ*, while the wall shear stress initially increases with the increase in *n* for opposing flow (*λ* < 0) for larger slip rate and it reduces for assisting flow (*λ* > 0). Fig 39 presents the variation of local Nusselt number for various values of *A*, *β* and *M*. It is noticeable that increasing values of *A* enhances the heat transfer rate whereas it slightly reduces with increase in *β* and *M*. The influence of for different values of *R*_*d*_, *δ* and Pr on the local Nusselt number is displayed in Fig 40. It is observed that heat transfer rate rises with increase in *R*_*d*_ and Pr whereas increasing values of *δ* decrease the heat transfer rate. Finally, Fig 41 presents the variation of the local Sherwood number for different values of *Sc*, *A* and *R*. It can be easily seen that Sherwood number, i.e., the mass transfer rate is higher for the increasing values of *Sc*, *A* and *R*.

## Conclusions

The objective of present study is to analyze unsteady hydromagnetic mixed convection flow of Casson fluid towards nonlinearly stretching sheet saturated in a porous medium under the influence of chemical and convective boundary conditions. The influence of viscous dissipation, joule heating, heat generation/absorption and partial slip is also considered in the present problem. The resulting governing equations are solved numerically using Keller-box method. The numerical as well as graphical results are obtained by developing an algorithm in MATLAB software. Physically, the effect of local unsteadiness parameter *A*, Casson parameter *β*, nonlinear stretching parameter *n*, magnetic parameter *M*, porosity parameter *K*, thermal buoyancy parameter *γ*, buoyancy ratio parameter *N*, Prandtl number Pr, radiation parameter *R*_*d*_, Eckert number *Ec*, heat generation/absorption parameter *ε*, Schmidt number *Sc*, chemical reaction parameter *R*, slip parameter *δ* and Biot numbers *Bi*_1_, *Bi*_2_ on fluid velocity, temperature, concentration as well as wall shear stress, heat and mass transfer rates are investigated. The main findings of this study can be summarized as follows:

1. The magnitude of wall shear stress, heat and mass transfer rates are found to be enhanced with the increase in *A*.
2. For increasing values of *β*, the velocity boundary layer becomes thinner whereas both thermal and concentration boundary layer become thicker with increase in *β*.
3. The fluid velocity reduces in the case of opposing flow (*λ* < 0) while enhances in the case of assisting flow (*λ* > 0) and opposite to this is observed for temperature and concentration distributions.
4. The fluid velocity is observed as increasing function of *R*_*d*_ when *λ* > 0 whereas decreasing function of *R*_*d*_ when *λ* < 0 and has no effect on velocity when *λ* = 0.
5. The velocity as well as concentration boundary layer thicknesses are noticed to be decreased with increase in *R*.
6. The small variations of temperature and concentration distributions are observed for the effect of *M* and *K* in the case of unsteady flow.
7. The variation of temperature for increasing values of *ε* is more pronounced for the steady flow.
